# Publisher Correction: A predictive internet-based model for COVID-19 hospitalization census

**DOI:** 10.1038/s41598-021-96363-y

**Published:** 2021-08-19

**Authors:** Philip J. Turk, Thao P. Tran, Geoffrey A. Rose, Andrew McWilliams

**Affiliations:** 1 Center for Outcomes Research and Evaluation, Atrium Health, Charlotte, NC 28204 USA; 2 Psychology Department, Colorado State University, Fort Collins, CO 80523 USA

Correction to: *Scientific Reports* 10.1038/s41598-021-84091-2, published online 03 March 2021

The original version of this Article contained errors in Table 1, where the first row was incorrectly formatted as a heading. The incorrect and correct values appear below.

Incorrect:
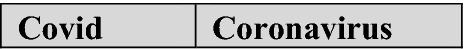


Correct:

**Table:** 

covid	coronavirus

The original Article has been corrected.

